# An Appeal to Medical Journal Editors: The Need for a Full Description of Laboratory Methods and Specimen Handling in Clinical Study Reports

**DOI:** 10.3109/00365513.2011.654052

**Published:** 2012-02-17

**Authors:** Nader Rifai, Thomas M Annesley, Jens P Berg, Carlo Brugnara, Edgard Delvin, Edmund J Lamb, Paul M Ness, Mario Plebani, Mark R Wick, Alan Wu, Joris Delanghe

**Affiliations:** 2 Clinical Chemistry; 3 Scandinavian Journal of Clinical & Laboratory Investigation; 4 American Journal of Hematology; 5 Clinical Biochemistry; 6 Annals of Clinical Biochemistry; 7 Transfusion; 8 Clinical Chemistry and Laboratory Medicine; 9 American Journal of Clinical Pathology; 10 Clinica Chimica Acta

We, the editors of the laboratory medicine journals, urge our colleagues in the International Committee of Medical Journal Editors (ICMJE)^1^ to adopt and enforce the following requirements for clinical studies that include laboratory testing for biomarkers, and to add these statements to the information for authors:

1. For commercial diagnostic tests, authors must include the actual name and generation of assay, the manufacturer, and the instrument used for analyses.
2. Authors must report performance characteristics, such as the imprecision of the assay in the investigators' laboratories, the assay's reportable range, and any reference (normal) range used in the study.
3. Authors must clearly indicate the types of specimens analyzed and the storage conditions for these specimens.

The rationale for these requirements is provided below.

Biochemical markers (here referred to as biomarkers) are being used increasingly in the diagnosis, staging, and prognosis of various diseases, as well as in the monitoring of response and determination of compliance with prescribed treatment regimens. Although a biomarker initially is developed and approved by regulatory agencies for a particular clinical use, other potential clinical applications of that biomarker usually evolve over time (e.g., cardiac troponin, B-type natriuretic peptide, C-reactive protein). These new potential applications often require the manufacturer to develop a more analytically sensitive or specific assay to meet the new clinical needs. It is common to have multiple generations of assays for a particular biomarker in the marketplace concurrently (cardiac troponin, thyroid-stimulating hormone). With the recent wave of mergers and acquisitions in the in vitro diagnostics industry, a single manufacturer can have multiple platforms and may feel obligated to have a similar menu of testing on all of them. In many cases, the same reagents for a given biomarker measurement may end up being used on several instruments that yield different results. Standardization and harmonization studies are directed at addressing these concerns to assure that the same result can be obtained for a biomarker, regardless of the method used for its measurement; however, such efforts are difficult, time-consuming, costly, and in some cases simply not feasible (e.g., measurement of fibrinogen by a functional assay). For many of the new biomarkers, standardization activities either are nonexistent or have been initiated but not finished. Therefore, it is crucial for authors of scientific reports to provide detailed information regarding the methods used in their studies for the measurement of biomarkers and for journal editors to demand such information so that the reader can interpret the findings.

In 2003, a group of scientists and editors developed the Standards for Reporting of Diagnostic Accuracy (STARD) statement, which was published in several laboratory and medical journals [1]. These guidelines, meant to improve the reporting and quality of studies of diagnostic accuracy, were endorsed in 2006 by the ICMJE (http://www.icmje.com). The STARD statement contained a checklist of 15 important items of information for authors to provide and for editors to require. Item no. 8 refers to the importance of describing in full the laboratory methods used and their performance characteristics. Similar recommendations were also made by REMARK for the reporting of tumor marker prognostic studies (item no. 5) [2]. Despite the obvious importance of the information recommended by these guidelines, however, progress in improving published reports on diagnostic accuracy has been modest [3]. Unfortunately, it remains common for descriptions of laboratory methods to be either incomplete or absent in reports describing sophisticated clinical studies that are published in excellent medical journals. The laboratory medicine journal editors would like to urge their medical journal editor colleagues to play a more active role in requiring a full description of laboratory assays used in clinical reports, and we provide these editors with 2 examples to illustrate the importance of disclosing such information.

## Example I

Cardiac troponins I (cTnl) andT (cTnT) have been recognized as the biomarkers of choice for the detection of myocardial injury and in the diagnosis of myocardial infarction and risk stratification of patients presenting with symptoms of acute coronary syndrome. Laboratory and clinical professional societies endorsed the use of the 99th-percentile value derived from a reference population as the clinical-decision threshold [4, 5].

At the present time, there are 23 cardiac troponin assays available (21 for cTnl, 2 for cTnT) from 14 manufacturers [6]. Four of these assays are for research use only, but data about their clinical utility have already made their way into the literature. Depending on their analytical sensitivity and imprecision characteristics, contemporary and high-sensitivity cardiac troponin assays are classified into one of 4 categories [6]. Most cTnl assays use antibodies that are unique to the manufacturer and recognize different antigenic sites [7]. Therefore, different manufacturers' assays measure different isoforms and/or fractions of the cTnl molecule [8]. As a result, the various cTnl assays yield different results for the same clinical sample; standardization efforts for cTnl assays have been hampered by these difficulties. A single manufacturer may have multiple cTnl assays on several analyzers with different performance characteristics. For example, Siemens has 5 cTnl assays on 5 different analyzers that yield 99th-percentile values ranging from 40 to 200 ng/L, despite the fact that they are all classified as contemporary assays [6]. Beckman Coulter has 2 cTnl assays, a first-generation assay and a third-generation high-sensitivity assay, which have 99th percentiles of 40 ng/L and 8.6 ng/L, respectively [6].

It is impossible for the reader to fully appraise the content, value, impact, and generalizability of a clinical study that utilizes cardiac troponin without knowing the exact cardiac troponin assay used and its performance characteristics. It is equally impossible for the researcher to perform a valid metaanal-ysis study assessing the utility of cardiac troponin in a particular clinical scenario without knowing the assays used in each of the studies included in the metaanalysis. Such information ensures that only data from comparable troponin assays are pooled.

## Example II

Although prostate-specific antigen (PSA) is used for the early detection of prostate cancer, its concentration is increased in patients with prostatitis and benign prostate hyperplasia. The measurement of free PSA (fPSA) and the determination of the ratio of fPSA to total PSA (tPSA), the so-called percentage of fPSA (%fPSA), helps to reduce the false-positive rate, thus avoiding unnecessary biopsy [9]. A variety of algorithms to improve prostate cancer risk prediction by combining age, tPSA, %fPSA, direct rectal-examination results, and/or prostate volume have been developed with data from different populations, by applying various tPSA intervals, and by using different manufacturers' PSA assays [10].

Although interassay variability among commercially available methods for tPSA and fPSA has improved as a result of using WHO PSA reference materials and developing equimolar-response assays, harmonization among methods is poor, and the interchangeability of tPSA, fPSA, and %fPSA results obtained by various assays is inadequate [11]. As a result, the diagnostic accuracy of prostate cancer risk prediction using a particular risk algorithm depends on the tPSA and fPSA assays used [12]. In addition, not considering PSA between-method differences [13] can lead to questionable conclusions regarding the performance of prediction tools of prostate cancer [14]. Therefore, it is imperative that the authors of such reports provide sufficient information regarding the methods used, to enable the reader to correctly evaluate the presented work and to be able to compare it to previously published studies.

Furthermore, every assay or analytical technique used in a study requires a specimen. Therefore, the type of specimen analyzed (e.g., serum vs plasma), type of blood-collection tube used (anticoagulant), storage conditions (refrigerated/frozen, temperature and duration of storage before analysis, freeze/thaw cycles), and analyte stability (especially for new biomarkers) may turn out to be important to the interpretation of results or the ability of others to reproduce the study. The reference range and the analytical reproducibility (imprecision) may also be assay-to-assay and site-to-site dependent, and therefore should be reported.

Prior to the electronic-publishing era, authors had to communicate their data and thoughts within strict word-limit constraints, with no other option for communicating additional information. Therefore, it is understandable that they kept their descriptions of laboratory methods to a minimum and elaborated on their findings. With the increasing availability of electronic supplements, however, authors have a mechanism to provide all needed information regarding the laboratory methods used in a study, thus enabling readers to fully evaluate the work. We sincerely hope that medical journal editors see the value of this argument and insist on having this information available to the reader. This relatively minor step will go a long way toward improving the quality of reporting the use of biomarkers in diagnostic studies.

This article is simultaneously being published in *Clinical Chemistry, Scandinavian Journal of Clinical and Laboratory Investigation, American Journal of Hematology, Clinical Biochemistry, Annals of Clinical Biochemistry, Transfusion, Clinical Chemistry and Laboratory Medicine, American Journal of Clinical Pathology*, and *Clinica Chimica Acta*.

## Footnotes

1 Nonstandard abbreviations: ICMJE, International Committee of Medical Journal Editors; STARD, Standards for Reporting of Diagnostic Accuracy; cTnl, cardiac troponin I; PSA, prostate-specific antigen; fPSA, free PSA; tPSA, total PSA; %fPSA, percentage of fPSA.
